# Shaping Workflows in Digital and Remote Diabetes Care During the COVID-19 Pandemic via Service Design: Prospective, Longitudinal, Open-label Feasibility Trial

**DOI:** 10.2196/24374

**Published:** 2021-04-05

**Authors:** Katarina Braune, Karina Boss, Jessica Schmidt-Herzel, Katarzyna Anna Gajewska, Axel Thieffry, Lilian Schulze, Barbara Posern, Klemens Raile

**Affiliations:** 1 Charité – Universitätsmedizin Berlin Department of Paediatric Endocrinology and Diabetes Berlin Germany; 2 Berlin Institute of Health Berlin Germany; 3 #dedoc° Diabetes Online Community Dedoc Labs Berlin Germany; 4 Novo Nordisk Center for Biosustainability Technical University of Denmark Copenhagen Denmark; 5 Designit Germany Berlin Germany

**Keywords:** telemedicine, telehealth, remote care, digital care, type 1 diabetes, pediatric diabetes, open source, service design, digital health, COVID-19, diabetes, workflow

## Abstract

**Background:**

The COVID-19 pandemic poses new challenges to health care providers and the delivery of continuous care. Although many diabetes technologies, such as insulin pumps and continuous glucose monitors, have been established, the data from these devices are rarely assessed. Furthermore, telemedicine has not been sufficiently integrated into clinical workflows.

**Objective:**

We sought to remotely support children with type 1 diabetes and their caregivers, enhance the clinical outcomes and quality of life of children with diabetes, increase multiple stakeholders’ engagement with digital care via a participatory approach, evaluate the feasibility of using an interoperable open-source platform in a university hospital setting, and analyze the success factors and barriers of transitioning from conventional care to digital care.

**Methods:**

Service design methods were used to adapt clinical workflows. Remote consultations were performed on a monthly and on-demand basis. Diabetes device data were uploaded from patients’ homes to an open-source platform. Clinical and patient-reported outcomes were assessed before, during, and after the COVID-19 lockdown period in Germany.

**Results:**

A total of 28 children with type 1 diabetes and their caregivers enrolled in this study and completed 6 months of remote visits. Of these 28 participants, 16 (57%) also opted to attend at least one of their regular visits remotely. After 3 months of remote visits, participants’ time in range (P=.001) and time in hyperglycemia (P=.004) significantly improved, and their time in hypoglycemia did not increase. These improvements were maintained during the COVID-19 lockdown period (ie, between months 3 and 6 of this study). Participants’ psychosocial health improved after 6 months.

**Conclusions:**

Remote consultations and commonly shared data access can improve the clinical outcomes and quality of life of children with type 1 diabetes, even during challenging circumstances. A service design approach helped with the delivery of comprehensive and holistic solutions that accounted for the needs of multiple stakeholders. Our findings can inform the future integration of digital tools into clinical care during and beyond the pandemic.

**Trial Registration:**

German Clinical Trials Register DRKS00016170; https://tinyurl.com/skz4wdk5

## Introduction

People’s interest in digital and remote care has been increasing worldwide. Although previous studies have demonstrated the effectiveness and increasing acceptability of telemedicine [1], knowledge on the implementation of digital care in health care settings and workflows remains limited.

The COVID-19 pandemic has forced many health care teams to find alternative approaches for delivering care to patients with chronic conditions. The demand for acute and emergency care has dramatically increased, and other areas of health care have been considerably compromised [2]. This is particularly concerning, as people with chronic conditions may have a higher risk for hospitalization and morbidity when they contract COVID-19. As such, health care professionals (HCPs) and public health institutions recommend that people who are at risk of SARS-CoV-2 infection should be protected from potential exposure to the virus. New legal frameworks that encourage health care teams to conduct remote, web-based consultations and prescribe medical software or apps have been introduced [3].

Diabetes is highly relevant to the field of telemedicine [4,5]. Intensive diabetes management has proven to be beneficial in delaying the onset of diabetes-related complications and reducing the severity of long-term complications [6]. Therapeutic guidelines have recommended a target hemoglobin A_1c_ (HbA_1c_) level of <7.0% (ie, <53 mmol/mol) for people with type 1 diabetes [7,8]. However, many people with type 1 diabetes cannot meet these target HbA_1c_ levels with standard care alone [9]. Modern treatment devices, such as insulin pumps and continuous glucose monitoring (CGM) sensors, are available to and widely used by people with diabetes in most industrialized countries. The uptake of insulin pumps and CGM sensors is highest in Western European countries [10-12] and the United States [9]. However, although less than a half of the population of people with type 1 diabetes in these regions use insulin pumps, the uptake of insulin pumps is much higher among children and adolescents. Germany, Switzerland, Luxembourg, and Austria have some of the highest technology uptake rates worldwide. For example, 92% of preschoolers in these countries used insulin pumps in 2017 [11]. The uptake of technology however decreases with age. Furthermore, although CGM sensors have been used by less than half of the population with diabetes (ie, typically young people with diabetes), CGM sensor uptake has increased considerably within the last 5 years in Germany and the United States [11,12]. However, to fully benefit from these relatively modern treatment options, people with diabetes require a high level of self-management, diabetes care teams require expertise and training, and appropriate digital infrastructures must be available in health care settings.

Value-based and integrated care are promising strategies for managing chronic conditions, as they emphasize the importance of shared decision making and the organization of care. These strategies are largely information-driven processes. Technological tools for collecting and exchanging information are essential to all stakeholders involved in integrated care [13,14]. From a patient’s perspective, integrated care aims to meet their health and social needs by using patient data as a starting point for redesigning their health care experiences.

Due to people’s interest in the transition from traditional care to digital care, which has considerably increased as a result of the COVID-19 pandemic, evidence related to HCPs’, patients’, and caregivers’ experiences with this transition has been emerging in various fields of medicine [15-28]. A variety of health care providers have shown increasing interest in the possibilities of digital health. However, there is little research on methods for integrating digital health tools into existing structures and workflows.

The Digital Diabetes Clinic (DDC) project sought to (1) remotely support children with type 1 diabetes and their caregivers during diabetes management; (2) increase the time that these children spend in the optimum glucose range (ie, time in range); (3) improve these children’s quality of life; (4) increase multiple stakeholders’ engagement with digital care via a participatory project that involves patients, caregivers, and care teams alike; (5) evaluate the feasibility of using an interoperable open-source platform to upload, store, and review diabetes device data in a university hospital setting; and (6) analyze the success factors and barriers of transitioning from conventional care to digital care.

## Methods

### Setting

**Figure 1:**
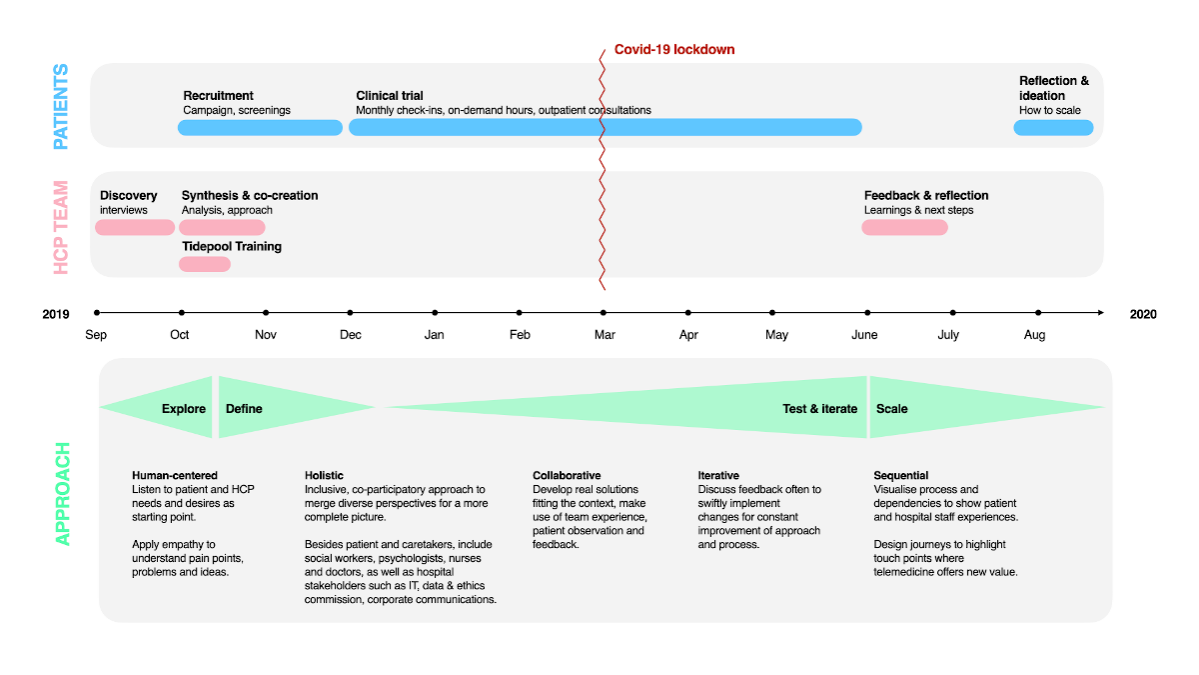
An overview of the methods that were used in this study; the interventions that involved the HCP team, patients, and caregivers; and the service design thinking approach. This is presented on a project timeline. HCP: health care professional; IT: information technology.

This study was conducted in a tertiary, multidisciplinary pediatric diabetes care center of a university hospital. All participating HCPs were actively attending to children and adolescents with diabetes, and all participating families were receiving diabetes care from the care center. An overview of the methods that were used in this study and the clinical trial design is shown in Figure 1.

### Service Design: A New Approach in Health Care

Service design is a strategic approach that is typically used in business environments and public sectors. This approach is used to create or improve processes for delivering desirable, consistent, and seamless experiences to users [29-31], such as staff, patients, and caregivers in health care settings. Service design thinking methods are used to assess the interrelations among different people, groups, organizations, resources, technologies, processes, and communication paths. This systemic and process-driven view is useful to design and deliver valuable services to users, including patients, their caregivers and health care professionals. Physical and digital touchpoints and resources are identified and evaluated, including data capture, usage and storage, as well as benefits and disadvantages of various communication channels (eg, apps, emails, phone calls, and written documentation).

### Workflow Analysis and Patients’ Journeys

**Figure 2:**
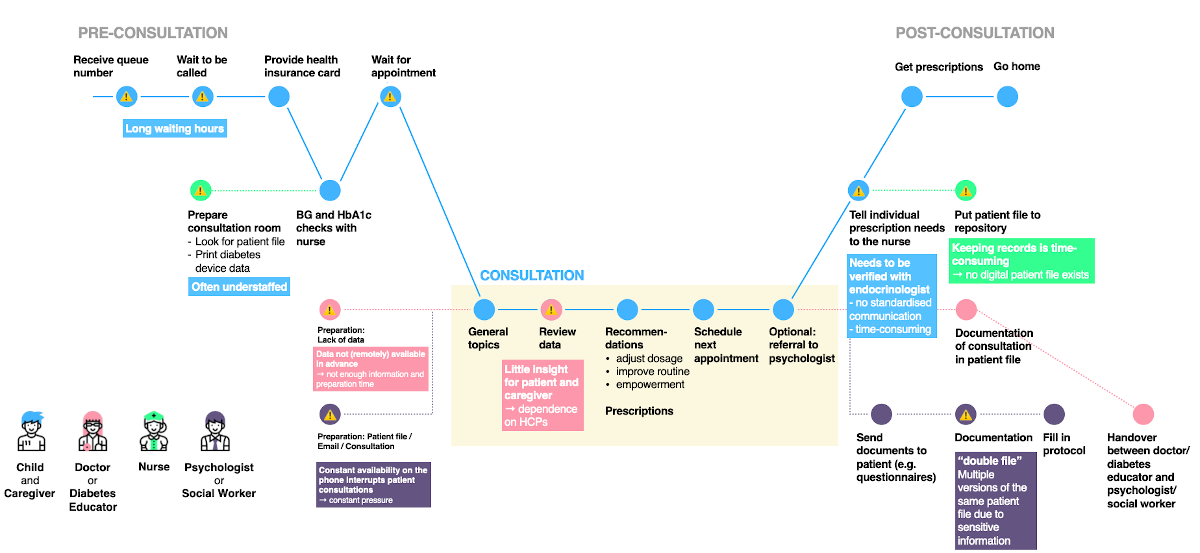
The "as-is" patient journey, the workflow of a conventional clinic visit, and current problem areas that require improvements. HbA_1c_: hemoglobin A_1c_; HCP: health care professional; BG: blood glucose.

**Figure 3:**
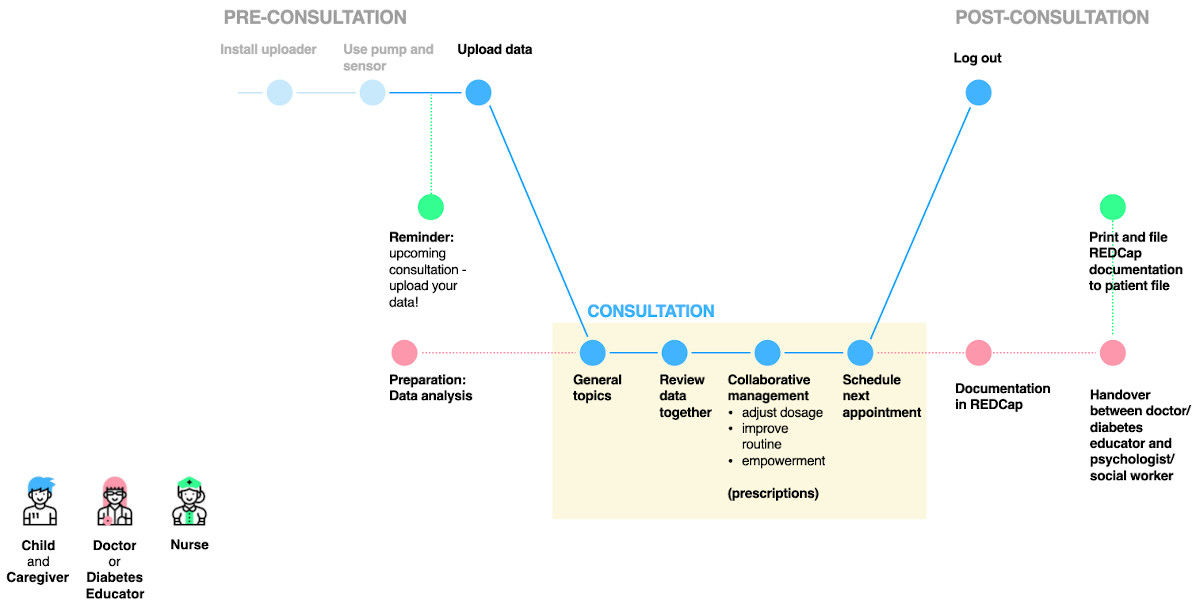
The "future" patient journey and the workflow of remote monthly check-ins. REDCap: Research Electronic Data Capture.

**Figure 4:**
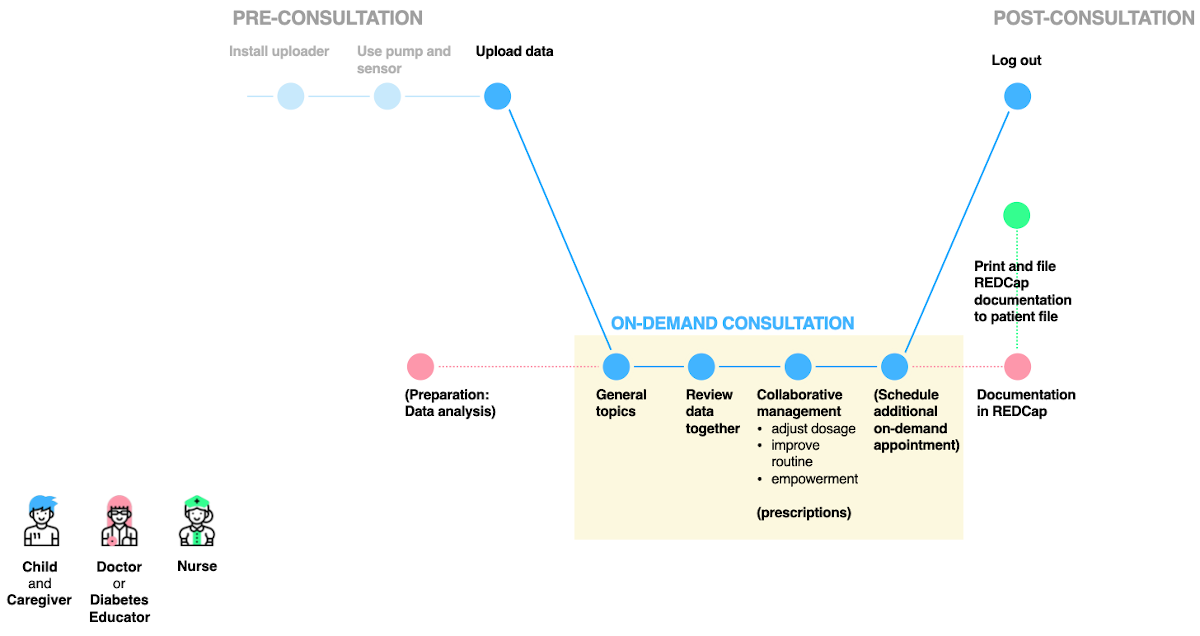
The patient experience and the workflow of remote, on-demand consultations (ie, those conducted between monthly check-ins). REDCap: Research Electronic Data Capture.

We followed the service design thinking process, starting with qualitative interviews. These interviews were conducted to assess users’ experiences (ie, journeys) with receiving pediatric diabetes care from a university hospital and identify problem areas (ie, pain points), such as moments of frustration and challenges/gaps in the care process. The design team conducted semistructured interviews with the health care team (ie, physicians, diabetes educators, nurses, social workers, and psychologists) via an exploratory approach to learn about existing workflows, group-specific wishes, and stakeholders’ needs. The interviews were conducted on site (ie, at the hospital) and in person. Each interview involved a small group, which included 2 researchers and 2-4 interviewees. Voice recordings, notes, and photographs of the hospital workspace allowed us to better understand and portray technology use in the university hospital. Interviewees and interviewers used post-its and sketches to depict the process/steps that patients and caregivers undergo during hospital visits, such as scheduling appointments, waiting for consultations, waiting for laboratory test results, and receiving prescriptions. This information was used to map a comprehensive patient journey. The as-is journey is used to identify areas for improvements, by taking into account workflows and perspectives of various stakeholders (Figures 2-4).

Using findings from the as-is journey, a future journey was created that offers a better patient experience. Afterward, we developed a strategic concept with a clear value proposition, which showed how different stakeholders can better coordinate their activities so that users can access new or improved services [32,33].

Once the concept was defined, we progressed to the implementation phase, during which the care team transitioned to their new responsibilities, adopted new technologies, and changed their routines and communication methods. This phase involved upskilling certain actors and transparently communicating these changes to patients, caregivers, care team members, organization members, or the general public.

### Stakeholder Workshops

A series of interactive workshops were held in preparation for and throughout the project. Initially, these workshops involved in-person meetings. Later, we transitioned to web-based meetings due to the COVID-19 pandemic. A participatory approach was used to facilitate a process for creating team trust; enabling clear communication; and promoting alignment, commitment, and shared goals. Relevant information about the project proposal was shared and discussed in order to identify concerns and expectations. Attendees were able to provide feedback and contribute to the design and organization of this study. The following questions were discussed: (1) “what do the terms ‘telemedicine’ and ‘digital care’ mean to us”; (2) “what are the pros and cons of a health care service that is carried out remotely”; (3) “how can health care settings, like a university hospital, adopt digital tools, new processes, and communication patterns into their current workflows”; (4) “which processes have to be adapted or redesigned”; (5) “what roles and responsibilities exist among the stakeholders”; and (6) “what technical and organizational support is needed”?

After the successful completion of 6 months of remote visits, a group of caregivers participated in a web-based workshop to share what they learned, discuss their reflections, and provide feedback. Similar to the previous workshops, our intention was to facilitate a participatory, cocreative session, during which caregivers could safely and comfortably voice opinions on how to improve users’ experiences with remote care. The study team created the following research questions: (1) “what was the study’s greatest benefit for you and your child”; (2) “if you could, what would be the one thing you would change”; (3) “what are the advantages or disadvantages of the DDC”; (4) “what elements should be adopted by existing health care services”; (5) “what challenges or benefits did you experience”; (6) “how did the experience impact the children”; and (7) “did they give any feedback on the new process”?

### Clinical Trial Design

A prospective, longitudinal, open-label feasibility trial was conducted at a single clinical center from December 2019 to June 2020. Children with type 1 diabetes who were aged 3-12 years were enrolled. To be included in this trial, the child had to live with a caregiver who could upload data and attend remote visits. Moreover, the treatment at the time of the initiation visit had to include the use of an insulin pump for at least 6 months and the use of CGM sensor for at least 1 month. Children were asked for their consent to continue CGM during this study. Caregivers’ ability to use an insulin pump and CGM sensor was verified. This included their ability to insert the infusion set, change the reservoir or patch pump (ie, where applicable), calibrate CGM sensors, and read and interpret related data.

Families could not participate in the trial if the caregivers who operated the study tools were diagnosed with a physical or mental health condition that severely interfered with their ability to complete the study protocol. Families were also excluded if they had no access to a computer or if they were experiencing problems with their at-home cellular/Wi-Fi connection (ie, problems that interfered with their ability to upload data and attend video consultations).

### Recruitment

We made families aware of this study by placing posters in the hospital’s waiting area, mailing informational materials to all families with children that fulfilled the inclusion criteria, and directly contacting families’ pediatric endocrinologists. Additionally, a website was launched to disseminate information on this study’s overall goals, activities, technicalities, and recruitment process. Eligible participants were invited to workshops in small groups. During these workshops, families learned about the study design and tools, provided their informed consent, and enrolled in this study.

### Hardware

Webcams with integrated microphones and speakers were provided to all participants. Caregivers could also use their own computers, tablets, or smartphone cameras. Access to a Windows personal computer or Macintosh computer with an internet connection was required to upload data.

### Data Platform

A personalized version of the open-source Tidepool platform (Tidepool Project) was used [34]. Caregivers could upload CGM sensor and insulin pump data to a secure and encrypted server that was hosted by Charité – Universitätsmedizin Berlin. They could also access their data via a web-based platform. As Tidepool is an open-source platform that is managed by a nonprofit group of parents of children with diabetes, its license permits third parties to use, reproduce, and alter the platform. Therefore, we were able to create a version of Tidepool that was adapted to the hospital’s information technology infrastructure and local data protection requirements [35-38]. This decision was based on the platform’s compatibility with multiple devices and manufacturers; our need to visualize CGM sensor and insulin pump data on a single platform in an integrated, device-agnostic fashion; the platform’s easy upload process; the care team’s and caregivers’ need for equal data access at all times; the need for a user interface that was simple to understand; and the availability of ambulatory glucose profiles with information on all important clinical outcome parameters.

Caregivers signed up for an account, installed the Tidepool uploader on their computer, and provided data access to the care team. All participants received training on how to upload and review data. Several participants also used other software, such as Dexcom Clarity (Dexcom Inc) and Abbott FreeStyle LibreView (Abbott Diabetes Care Inc).

### Remote Visits

A secure, web-based video chat app (ie, Patientus [Jameda Gesellschaft mit beschränkter Haftung]) was used to conduct remote visits. As previously agreed upon in the stakeholder workshops, remote visits with a certified diabetes educator were scheduled on a monthly basis. This ensured that a frequent number of follow-ups were conducted and limited the additional burden on participating families at the same time. In addition, participants were offered optional, daily, on-demand consultation hours. Participants could also opt to remotely attend their routine appointments (ie, those that occurred every 2-3 months) with their pediatric endocrinologists. These appointments typically involved in-person visits, which were a part of participants’ standard care.

### Outcome Measures

The primary outcome parameter was time in range, which refers to the percentage of time that participants spend with a sensor glucose level of 70-180 mg/dL (ie, 3.9-10.0 mmol/L).

Our secondary endpoints included the following: (1) time in hypoglycemia, which refers to the percentage of time that participants spend with a sensor glucose level of <54 mg/dL (ie, 3.0 mmol/L) and 54-70 mg/dL (ie, 3.0-3.9 mmol/L); (2) time in hyperglycemia, which refers to the percentage of time that participants spend with a sensor glucose level of >250 mg/dL (ie, 13.9 mmol/L) and 180-250 mg/dL (ie, 10.0-13.9 mmol/L); (3) the incidence of severe hypoglycemia presenting with the need for assistance from others or unconsciousness; (4) the incidence and suspected cause of diabetic ketoacidosis; (5) diabetes-related hospitalizations; (6) HbA_1c_ (ie, if available) and estimated HbA_1c_ levels; (7) participants’ quality of life, which parents reported via a web-based survey (ie, the Pediatric Quality of Life Inventory questionnaire); and (8) the feasibility of the care model, which was based on the proportion of participants who successfully complete 6 months of remote visits, as well as caregiver feedback from web-based surveys and interactive workshops (ie, optional feedback).

### Caregiver Feedback

At the end of the study, caregivers were invited to provide feedback via an optional web-based survey, which asked them to share the pros and cons of their experiences with remote care and their expectations and needs for future diabetes care.

### Data Collection and Analysis

A deidentified data set that included demographic data, outcome measures, and consultation information was documented and analyzed with the REDCap (Research Electronic Data Capture; Vanderbilt University) platform. REDCap is a secure electronic data capture tool that is hosted locally at Charité – Universitätsmedizin Berlin [39]. Furthermore, remote and in-person consultations were documented in the hospital’s information system. Quantitative analyses were conducted with the R version 4.0.2 programming framework (The R Foundation), and the ggplot2 package was used to generate figures. Changes in primary and secondary outcome parameters were assessed with the Wilcoxon signed-rank test, which included a P value threshold of .05 for paired data and a 1-tailed test with an alternative hypothesis (ie, “less”). With regard to HbA_1c_ descriptive statistics and associated statistical tests, missing HbA_1c_ values were substituted with estimated HbA_1c_ values (ie, when available).

### Ethical Conduct and Informed Consent

This study was conducted in accordance with the Declaration of Helsinki, Good Clinical Practice guidelines, and Data Privacy Law of Berlin (ie, the Berliner Datenschutzgesetz or Berlin Data Protection Act). This study was approved by the Charité ethics committee (approval number: EA2/125/18) and registered under the clinical trial registration number DRKS00016170. Informed consent was obtained from each child’s caregiver prior to a family’s inclusion in this study. A child-friendly version of the information sheet was provided to children aged 8-12 years, and they were asked for their assent to participate.

## Results

We present the results of a 6-month feasibility trial, information on the conceptual development of the trial, and details on the lessons that we learned throughout the process.

### Patient Journey and Workflow Analysis

The service design activities involved 5 pediatric endocrinologists, 3 diabetes educators, 3 nurses, 3 psychologists/social workers, 1 resident physician, and 4 families that previously participated in telemedicine interventions.

Figure 2 illustrates the patient journey and the current workflow of a conventional visit to the hospital. Multiple pain points were identified, such as long waiting hours; the lack of digitally available data from devices; patients’/caregivers’ limited insight into their own data; and time-consuming tasks for the care team, such as documentation processes and the provision of paper file logistics. In the proposed workflow for scheduled remote consultations (Figure 3) and on-demand remote consultations (Figure 4), the identified problem areas were addressed; HCPs and patients/caregivers were provided with equal access to data, and they collaboratively agreed upon individual therapy goals and methods for achieving them. The documentation process was simplified.

### Stakeholder Workshops

**Figure 5:**
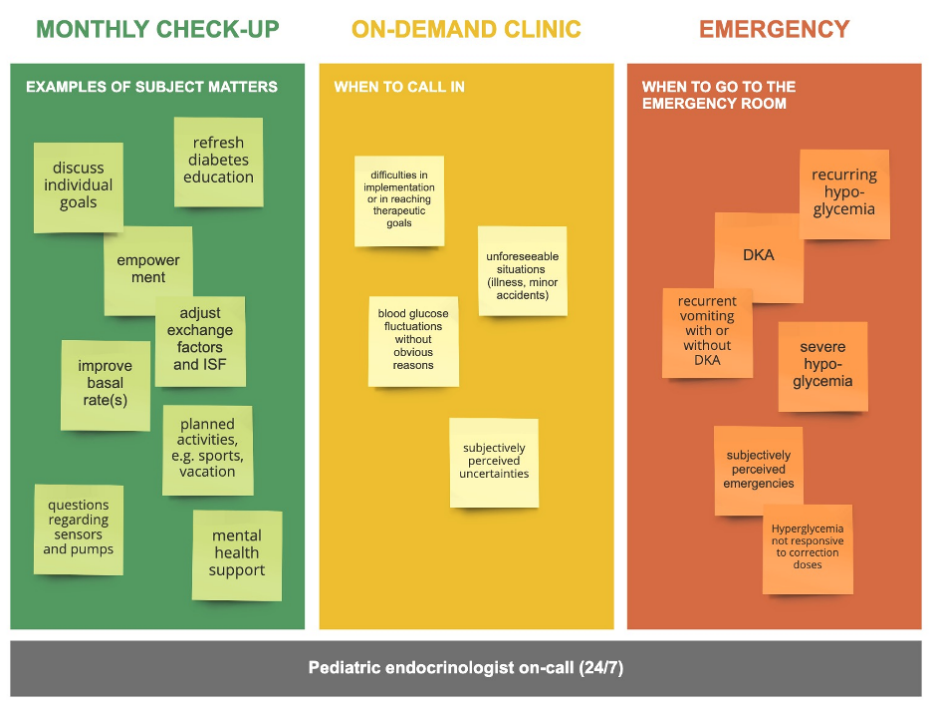
The triage system for on-demand consultations; risk assessments; and the emergency management of severe hypoglycemia, DKA, or other emergencies that are subjectively perceived as serious. DKA: diabetic ketoacidosis; ISF: insulin sensitivity factor.

The stakeholders agreed that the project’s value proposition was to improve patients’ well-being by using existing ambulatory services to complement remote consultations instead of completely replacing ambulatory services, and by improving data access and analysis. Much time was spent on structuring a remote service that promotes better interactions, higher flexibility, and lower stress among all stakeholders. The clear advantages of telemedicine were identified, such as the ability to save time and effort and the possibility of delivering health care to families in rural areas. Furthermore, people believed that digital care was less bureaucratic than nondigital care. Hence, digital care was less time-consuming than nondigital care, as per the documentation. Therefore, health care providers could save money by using digital care services. The care team was also generally open to the adoption of new technology, as they saw clear benefits for patients. Limited staff availability due to the increasing economization of the health care sector, organizational challenges, and structural challenges were perceived to be the main barriers to the adoption of new tools and care pathways. Furthermore, safety concerns were addressed and a triage system for emergency scenarios was created (Figure 5).

The perceived challenges that were identified in this study were mainly technical in nature. Such challenges included the compatibility between several sensors/pumps and the Tidepool uploader and hospital server errors that required time-consuming and complex solutions.

### Study Cohort

A total of 28 patients (age: median 8 years, SD 2.6 years) with diabetes (duration: median 4 years, SD 2.2 years) were enrolled in this study. It should be noted that 3 more families were interested in participating in this study, but they could not be included due to the incompatibility of their diabetes hardware (n=1), their lack of access to a computer (n=1), and technical issues with their at-home personal computers (n=1). Of the 27 participating families, 1 (4%) had 2 children with diabetes, 2 (7%) came from single-parent households, 2 (7%) had parents who lived separately, and 7 (26%) had a migration background (ie, at least 1 parent was born in a country other than their country of residence). All 28 children used insulin pumps and CGM sensors as a sensor-augmented pump therapy system (n=27) or hybrid closed-loop system (n=1). Families had a median annual household net income of €60,000 (US $72,824.10; SD €34,078 [US $41,361.70]), and 45% (28/62) of caregivers had a university degree. The income and education levels of the cohort were above the national average. Children’s CGM sensor and pump supplies were fully covered by their health insurance; 85% (23/27) of the families had a public health care plan and 15% (4/27) had a private health care plan. A summary of the cohort’s demographic characteristics are reported in Table 1.

**Table 1:** Sociodemographic characteristics of the study cohort.

Characteristics		Value
**Gender, n (%)**		
	Female	18 (64.2)
	Male	10 (35.7)
	Other	0 (0)
**Comorbidities, n (%)**		
	Celiac disease	3 (10.7)
	Hashimoto thyroiditis	1 (3.6)
	Other	2 (7.1)
**Type of insulin pump, n (%)**		
	Medtronic 670G	1 (3.6)
	Medtronic 640G	23 (82.1)
	Medtronic Veo	4 (14.3)
**Type of continuous glucose monitoring sensor, n (%)**		
	Medtronic Guardian	13 (46.4)
	Dexcom G6	5 (17.9)
	Dexcom G5	1 (3.6)
	FreeStyle Libre 2	9 (32.1)
**Caregiver’s/mother’s employment status, n (%)**		
	Full-time employment	10 (35.7)
	Part-time employment	13 (46.4)
	Unemployed	4 (14.3)
	Student	1 (3.6)
	Not available	0 (0)
**Caregiver’s/mother’s highest educational level, n (%)**		
	No/some high school	1 (3.6)
	High school	4 (14.3)
	University degree or diploma	12 (42.9)
	Doctorate	2 (7.1)
	Other	9 (32.1)
	Not available	0 (0)
**Caregiver’s/mother’s professional background, n (%)**		
	Health care/science	8 (28.6)
	Education and childcare	3 (10.7)
	Information technology	1 (3.6)
	Service	3 (10.7)
	Other	9 (32.1)
	None	1 (3.6)
	Not available	0 (0)
**Caregiver’s/father’s employment status, n (%)**		
	Full-time employment	22 (78.6)
	Part-time employment	2 (7.1)
	Unemployed	2 (7.1)
	Student	1 (3.6)
	Not available	1 (3.6)
**Caregiver’s/father’s highest education level, n (%)**		
	No/some high school	4 (14.3)
	High school	3 (10.7)
	University degree or diploma	13 (46.4)
	Doctorate	1 (3.6)
	Other	6 (21.4)
	Not available	1 (3.6)
**Caregiver’s/father’s professional background, n (%)**		
	Health care/science	2 (7.1)
	Education and childcare	1 (3.6)
	Information technology	4 (14.3)
	Service	2 (7.1)
	Other	14 (50)
	None	2 (7.1)
	Not available	1 (3.6)

### Feasibility

All enrolled participants completed 6 months of monthly remote visits. In addition, 57% (16/28) of the participants opted to remotely attend at least one of their regular clinic visits (ie, those that occurred every 2-3 months) with their pediatric endocrinologist. The on-demand clinic service was used by 29% (8/27) of the families. Of these 8 families, 7 (88%) made use of this service once, and 1 (12%) used this service multiple times throughout the study. The subject matters that were discussed during remote visits are summarized in Table 2. In 96.4% (118/122) of the consultations, participants felt confident with remotely uploading, accessing, and reviewing their data. Although the technical aspects of the data and video chat platforms were mostly discussed during the first web-based visit, follow-up visits largely focused on diabetes- and health-related topics. During the monthly check-ups, 90.4% (110/122) of children fully achieved their individual therapy goals, and 6.1% (7/122) partially achieved their individual therapy goals. There were no study dropouts. Severe hypoglycemia, diabetic ketoacidosis, or other issues that required study personnel to consult the on-call endocrinologist did not occur. Patient handovers to psychologists and social workers occurred 6 times.

**Table 2:** Activities and subject matters that were discussed during remote care visits.

Activities and subject matters	Monthly check-in, n (%)	On-demand clinic, n (%)
Reviewed data together	96 (85.7)	6 (60)
Basal rate adjustments	59 (52.7)	4 (40)
Carbohydrate exchange factor adjustments	52 (46.4)	3 (30
Technical aspects of the data platform	34 (30.4)	1 (10)
General organizational matters	32 (28.6)	1 (10)
Refreshed diabetes education and training	25 (22.3)	2 (20)
Technical aspects of the video chat platform	18 (16.1)	2 (20)
Mental health concerns	13 (11.6)	1 (10)
Technical aspects of continuous glucose monitoring	9 (8)	1 (10)
Technical aspects of insulin pumps	8 (7.1)	1 (10)
Therapy adjustments for physical activity	7 (6.3)	0 (0)
Insulin sensitivity factor adjustment	5 (4.5)	1 (10)
Acute illness	2 (1.8)	1 (10)
Other topics	30 (26.8)	3 (30)

### Clinical and Patient-Reported Outcomes

**Figure 6:**
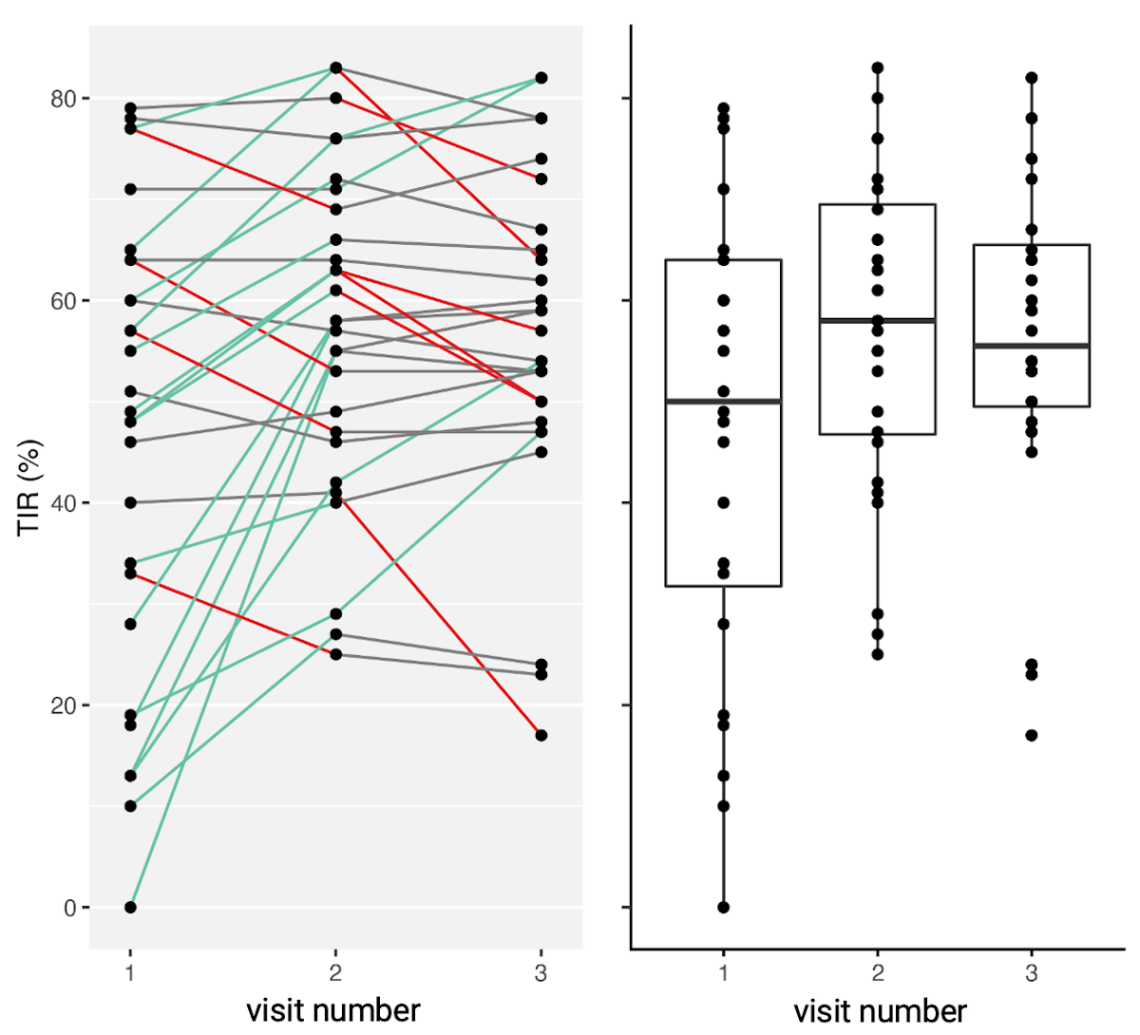
The individual changes and overall distribution of participants’ percent TIR of sensor glucose targets before remote consultations (ie, visit 1), after 3 months of remote consultations (ie, visit 2), and after 6 months of remote consultations (ie, visit 3). Green lines indicate that the individual change in TIR was >5% (ie, high amount of change). Red lines indicate that the individual change in TIR was <5% (ie, low amount of change). Grey lines indicate that the individual change in TIR was within 5% of the acceptable range (ie, a stable TIR). TIR: time in range.

After completing 3 months of remote consultations, participants’ time in range (P=.001) and time in hyperglycemia (P=.004) significantly improved (Figure 6). This improvement was maintained over the lockdown period (ie, between months 3 and 6 of this study); daycares, playgrounds, schools, universities, nonessential businesses, and international borders were closed during the first wave of the COVID-19 pandemic in Germany (ie, from March to May 2020). After 3 and 6 months of remote visits, patients’ time in hypoglycemia did not significantly increase (3 months: P=.21; 6 months: P=.08), no significant changes in HbA_1c_ levels were observed (3 months: P=.43; 6 months: P=.42), and patients’ psychosocial health significantly improved. All details on outcome parameters are shown in Table 3.

**Table 3:** Clinical and patient-reported outcomes before, during, and after 6 months of remote consultations. All significance levels for the visit 2 and visit 3 results were compared to those for visit 1.

Outcome measures		Visit 1 (ie, enrollment)		Visit 2 (ie, 3 months of completed remote consultations)		Visit 3 (ie, 6 months of completed remote consultations)	
		Value, mean (SD)	P value	Value, mean (SD)	P value	Value, mean (SD)	P value
Time in range, %		46.9 (22.9)	Referent	57.5 (16.3)	.001	56.3 (16.5)	.02
**Time in hypoglycemia (mg/dL [mmol/L]), %**		5.1 (12.1)	Referent	4.7 (6.9)	.21	3.7 (4.3)	.08
	54-70 (3.0-3.9)	3.1 (5.4)	Referent	3.5 (5.0)	.17	2.7 (3.0)	.11
	<54 (3.0)	2.0 (6.9)	Referent	1.2 (2.1)	.39	1.1 (1.4)	.12
**Time in hyperglycemia (mg/dL [mmol/L]), %**		48.0 (26.2)	Referent	37.9 (18.8)	.004	40.0 (18.7)	.02
	180-250 (10.0-13.9)	26.7 (14.4)	Referent	25.9 (13.2)	.35	26.1 (10.0)	.47
	>250 (13.9)	21.3 (20.2)	Referent	12.0 (12.8)	.004	13.9 (14.7)	.01
Hemoglobin A_1c_ level, %		7.5 (0.9)	Referent	7.7 (0.9)^a^	.43	7.6 (1.1)	.42
Hemoglobin A_1c_ level, mmol/mol		58.2 (9.0)	Referent	58.1 (9.3)^a^	.43	59.1 (13.0)	.42
**Quality of life score^b^**							
	Psychosocial health summary score	72.5 (14.0)	Referent	—^c^	—	78.0 (14.4)	.04
	Physical health summary score	81.0 (17.9)	Referent	—	—	82.7 (16.0)	.14
	Diabetes-related score	70.3 (10.7)	Referent	—	—	73.6 (9.0)	.06

^a^Not available for patients who opted to perform their month-3 visit remotely. Instead, estimated hemoglobin A_1c_ values were calculated based on sensor data.

^b^Based on Pediatric Quality of Life Inventory scores.

^c^Not available.

### Caregiver Feedback

Caregivers expressed that remote care was beneficial to them and their child. They also believed that remote care had advantages over in-person meetings. Reductions in the amount of time and stress (ie, those associated with hospital visits); flexibility during different times of the day; and the opportunity to be in a safe, comfortable, and familiar setting allowed for more engagement and dedicated interactions between families and health service providers. Families also believed that health service providers were less stressed, more dedicated, and focused. The ability to review data together resulted in new and valuable insights for most stakeholders and enabled caregivers to take initiative and make adjustments to therapy. The families were satisfied with the care that they received. They also expressed their desire to continue digital care after their participation in this study and suggested that digital care should be fully integrated into routine care. Furthermore, families desired remote consultations with psychologists and social workers. The intervals between visits were shorter, and caregivers perceived this as beneficial for discussing any arising questions. The late afternoon and evening hours were caregivers’ preferred times for attending consultations. Technical problems with internet connections, video chats, and data platforms occasionally occurred.

Finally, remote care was perceived to be “more modern, timely and suited to their needs,” with a perceived general improvement of the children’s well-being, improved glycemic outcomes, and newly gained insights. Details on caregiver feedback are shown in Table S1 in Multimedia Appendix 1.

## Discussion

### Principal Results

This study describes the feasibility of remote care, the transition from traditional care to digital care, and the use of an interoperable health data platform in an ambulatory pediatric diabetes care setting. Digital care was delivered successfully, and participants were satisfied with the care that they received. Remote and continuous data access considerably improved for patients, caregivers, and the health care team. Data access was perceived as helpful for therapeutic decision making. Despite the COVID-19 pandemic and its numerous potential implications for physical and mental health [40,41] and people’s social and family lives, better clinical outcomes were achieved before the intervention period, and these outcomes were maintained during the intervention period. Furthermore, patients’ psychosocial health significantly improved.

Our findings are in line with those of studies that were conducted before [4,5,42-44] and during [45-51] the COVID-19 pandemic (ie, studies that reported on the benefits of remote care in various regions and settings). Our study is the first to report on the following: (1) the impact that remote care during the COVID-19 pandemic has on clinical outcomes; and (2) the integration of remote care into pediatric diabetes care during the COVID-19 pandemic. Our study is also the first to describe how service design methods were used to increase stakeholder engagement and improve workflows in clinical care.

Conventional hospital visits and admissions provide patients with artificial time windows that rarely address their actual daily challenges and individual needs. Young people with diabetes and caregivers have often perceived a lack of communication with their health care providers when it comes to discussing their treatments [52]. They have also felt that their care teams do not sufficiently understand their daily problems and do not have a lot of involvement in the therapeutic decision-making process [52]. Studies have shown that few people with diabetes and caregivers regularly download and review their own data [53,54]. It has also been reported that self-assessments and specialists’ referrals (ie, those for obtaining data) can improve diabetes management [55].

The findings from this study allowed us to validate the technical instruments and organizational processes that are necessary for the transition to digital care and new workflows. Both the “as-is” journeys and “future” journeys were key tools for promoting shared understanding and alignment among stakeholders. As recent modalities have led to overall changes in work culture and social interactions (eg, the majority of professional meetings, social gatherings, university coursework, and school classes have been conducted via web-based platforms), there is a strong need for the health care sector to adopt new methods for communication. The implementation of remote data assessments and web-based communications can therefore improve people’s access to specialty care during and beyond the pandemic. However, a paradigm shift in the delivery, management, and funding of health care services is required.

The use of an open-source data platform was considered positive for the following reasons: (1) it was interoperable with devices from different manufacturers; (2) it allowed both parties to immediately access data; and (3) it provided a positive user experience. However, technical issues occasionally occurred throughout the intervention period. These issues were sometimes challenging to solve, as the data platform was not a plug-and-play service that was provided by a third party. Therefore, follow-up projects should include structured management plans that clarify the roles and responsibilities of technical support personnel.

During the transition to technological innovations, the digital divide might leave several user groups behind. Therefore, while people with diabetes have generally reported positive experiences with diabetes technology, the complexity of accessing and maintaining such technology remains a challenge. It should be noted that younger adults, who are generally perceived as “tech savvy,” are in fact less likely to embrace the use of diabetes technology. Additionally, physical barriers (eg, the need to carry devices) and general diabetes distress are more severe among younger adults than older adults [56].

In this study, the team primarily interacted with caregivers. Further research is needed on actively increasing children’s and adolescents’ involvement in telemedicine consultations and adapting digital care interventions to cater to the needs of other age groups, people with diabetes (ie, other than type 1 diabetes), and people who use self-monitoring blood glucose devices and metered dose inhalers (eg, uploading data from glucometers and electronic pens or integrating diabetes diary apps into routine care). Based on the stakeholder feedback, we conclude that it is crucial to integrate new digital tools into routine care instead of creating separate care pathways. Therefore, further service design research should address how health care teams in other health care settings (eg, high-volume clinics for adult diabetes care) and staff with limited technical skills can be trained to confidently provide digital care.

The ability to effectively provide telemedicine increases if a clinic has experience with diabetes technology provision (eg, insulin pumps, CGM sensors, electronic health records, diabetes management software, etc) and the required infrastructure (eg, software, computers, and internet connections). However, it is worth acknowledging that although the use of diabetes technology is clearly associated with countries’ local reimbursement strategies or health insurance plans [57], diabetes technology uptake is heterogeneous in countries that offer full reimbursements. Individual aspects such as personal attitudes or interests (ie, those of people with diabetes and, more importantly, HCPs), awareness, structure, and capacity are insurance-related determinants to improving the accessibility of diabetes technology [58]. Thus, we are aware that our proposed model may not be suitable for all pediatric diabetes clinics.

The beliefs and attitudes of HCPs may be barriers to increasing the universal accessibility of advanced diabetes technologies [56,59,60]. New care models may initially result in feelings of uncertainty, and they might seem overwhelming to people who have usually delivered in-person care for the past few decades [61]. Organizational and structural changes can lead to frustration and negatively impact people’s motivations for adopting new care pathways. To ensure that health care teams do not shy away from new technology and additional work requirements, all relevant stakeholders must be engaged with the transition process as early as possible. The business environment has learned that the service design approach can be used to innovate methods for addressing people's needs. Therefore, the health care ecosystem can greatly benefit from using the same approach [31]. As an applied research and innovation framework, service design prioritizes empathy for the users of a service or product; embraces interdisciplinarity and collaboration within project teams; and encourages the action-oriented, rapid prototyping of user-derived insights instead of top-down hypotheses. Service design has proven to be beneficial for encouraging all stakeholders to contribute their ideas during the design process, acknowledge their concerns, and build supportive practices [61].

Our study adds to the ongoing discussion on the importance of time in range (ie, as a measure that is comparable to or more important than HbA_1c_ levels). Our choice to use time in range as the primary outcome parameter and HbA_1c_ level as a secondary outcome parameter may be a strength of our study (ie, compared to most other studies that have reported on the clinical outcomes of people with diabetes). Although our study cohort’s time in range and quality of life significantly improved, there were no significant changes in patients’ HbA_1c_ levels. Although HbA_1c_ is widely used as a primary outcome parameter in many CGM studies [62-66], time in range has been acknowledged as an outcome measure for representing glycemic control [67,68]. Studies have only shown moderate correlations between time in range and associated HbA_1c_ levels (ie, changes in HbA_1c_ levels for a given change in time in range widely varies) [69]. Our findings support the fact that there is a need to use CGM-derived outcome measures in clinical care and clinical research and a need to identify new surrogate markers for the development of diabetes-related long-term complications [67-69]. These needs can be addressed, as CGM data can be remotely assessed without having to resort to additional invasive procedures for people with diabetes. Furthermore, clinical outcome improvements may remain unobserved if researchers only focus on analyzing HbA_1c_ levels.

We acknowledge that our study has several strengths and limitations. First, this study provides other diabetes care teams with a practical example of how to take advantage of the opportunities that have arisen from the necessity of remote care. Second, our study supports the use of technology in the delivery of diabetes care and the promotion of patient involvement in the cocreation of services. Third, our study is based on real-world data; it presents the different perspectives of health service providers and users. The limitations of this study should also be acknowledged. This study was a single-arm, nonrandomized feasibility trial that analyzed data from a small cohort of patients. Due to the trial’s observational nature and our lack of a control group, our ability to assess the effectiveness of remote and routine care for all people with diabetes was limited. Although our participants had widely varying characteristics (ie, various education levels, income levels, and professional backgrounds), the majority of the participants were from middle- to high-income and educated families, and all participants used insulin pumps and CGM sensors. This may mean that socioeconomic status–related biases are present in our study. Furthermore, people who experience language barriers and people with low levels of technological literacy might not have felt confident with participating in this study. This might indicate that selection bias was present in our study. Additionally, access to the internet and a computer was required. Although these technologies are available to most families, they were not available to all eligible families. Furthermore, as CGM sensors, insulin pumps, and supplies are fully covered by the public and private health insurance plans in Germany (ie, for children with diabetes), this project may not be applicable to all diabetes care settings. Limited access to diabetes technologies, which is evident in many regions outside of Western Europe, could limit the applicability of our service design approach. Although advances in technologically mediated treatments are promising, there are still concerns about social inequality and the challenge of ensuring that such treatments are widely disseminated across the population. More research is needed to understand these potential obstacles and provide appropriate education and support.

### Conclusions

This study sought to identify and solve the following problems in diabetes care: the limited accessibility of diabetes device data; the poor interoperability of data from different devices; and restricted access to specialists, especially during a global pandemic. Our study design allowed the care team, patients, and caregivers to actively contribute to the DDC project and promoted shared decision making. The results generated by this study will help to inform and improve methods for implementing remote and digital diabetes care into the wider health care sector during and beyond the pandemic.

## Appendix

Multimedia Appendix 1 Table S1. Caregiver feedback regarding their experience with remote consultations.

## Abbreviations

CGM: continuous glucose monitoring
DDC: digital diabetes clinic
HbA_1c_: hemoglobin A_1c_
HCP: health care professional
REDCap: Research Electronic Data Capture
