# Supplementary material for: Shaping Workflows in Digital and Remote Diabetes Care During the COVID-19 Pandemic via Service Design: Prospective, Longitudinal, Open-label Feasibility Trial
Source: JMIR Mhealth Uhealth. 2021 Apr 5;9(4):e24374. doi: 10.2196/24374 (PMC8023381; doi:10.2196/24374)
Supplement: Multimedia Appendix 1 [file mhealth_v9i4e24374_app1.docx]

**Suppl. Table 1:** Caregiver feedback regarding their experience with remote consultations

| **Perceived changes** | **N [%]** |
| --- | --- |
| General improvements by participation in the Digital Diabetes Clinic         Yes         No | 18 (85.7)  3 (14.3) |
| General worsening by participation in the Digital Diabetes Clinic        Yes        No | 0 (0)  21 (100) |
| Advantages of remote consultations vs. in-person meetings       Yes       No | 21 (100)  0 (0) |
| Disadvantages of remote consultations vs. in-person meetings       Yes       No | 11 (52.4)  10 (47.6) |
| Satisfaction with medical care     Fully satisfied     Somewhat satisfied     Neither satisfied nor dissatisfied     Somewhat dissatisfied     Dissatisfied | 17 (81.0)  4 (19.0)  0 (0)  0 (0)  0 (0) |
| Satisfaction with video chat platform     Fully satisfied     Somewhat satisfied     Neither satisfied nor dissatisfied     Somewhat dissatisfied     Dissatisfied | 7 (33.3)  10 (47.6)  3 (14.3)  1 (4.8)  0 (0) |
| Satisfaction with data platform     Fully satisfied     Somewhat satisfied     Neither satisfied nor dissatisfied     Somewhat dissatisfied     Dissatisfied | 8 (38.1)  4 (19.0)  5 (23.8)  3 (14.3)  1 (4.8) |
| Satisfaction with schedule of appointments     Fully satisfied     Somewhat satisfied     Neither satisfied nor dissatisfied     Somewhat dissatisfied     Dissatisfied | 15 (71.4)  4 (19.0)  2 (9.5)  0 (0)  0 (0) |
| Satisfaction with appointment duration     Fully satisfied     Somewhat satisfied     Neither satisfied nor dissatisfied     Somewhat dissatisfied     Dissatisfied | 19 (90.5)  2 (9.5)  0 (0)  0 (0)  0 (0) |
| Likelihood to continue with remote care     Very likely     Likely     Neither likely nor unlikely     Rather unlikely     Unlikely | 18 (85.7)  3 (14.3)  0 (0)  0 (0)  0 (0) |
| Data platforms used     Tidepool     Dexcom Clarity     Abbott Libre View     Other | 21 (100)  4 (19.0)  7 (33.3)  0 (0) |
| Technical problems with the internet connection occurred    Always    Often    Sometimes    Rarely    Never | 0 (0)  0 (0)  3 (14.3)  7 (33.3)  11 (52.4) |
| Technical problems with hardware and software of the computer occurred    Always    Often    Sometimes    Rarely    Never | 0 (0)  1 (4.8)  3 (14.3)  8 (38.1)  9 (42.9) |
| Technical problems with video chat occurred    Always    Often    Sometimes    Rarely    Never | 0 (0)  1 (5.0)  5 (25.0)  13 (65.0)  1 (5.0) |
| Technical problems with data uploader occurred    Always    Often    Sometimes    Rarely    Never | 0 (0)  3 (15.0)  6 (30.0)  9 (45.0)  2 (10.0) |
| Technical problems with data platform occurred    Always    Often    Sometimes    Rarely    Never | 0 (0)  1 (4.8)  3 (14.3)  13 (61.9)  4 (19.0) |
| Problems with interpreting the data    Always    Often    Sometimes    Rarely    Never | 0 (0)  0 (0)  4 (19.0)  6 (28.6)  11 (52.4) |
| Gathered new insights into diabetes by looking at the data    Yes    No | 15 (71.4)  6 (28.6) |
| Remote consultations were helpful to adjust therapy based on the data    Yes    No | 21 (100)  0 (0) |
| Pandemic had a notable influence on diabetes management    Yes    No | 15 (71.4)  6 (30.0) |
| Homeschooling instead of school/daycare during the lockdown    Always    Often    Sometimes    Rarely    Never | 3 (14.3)  9 (42.9)  6 (28.6)  0 (0)  3 (14.3) |
| No physical activity    Always    Often    Sometimes    Rarely    Never | 5 (23.8)  6 (28.6)  5 (23.8)  3 (14.3)  2 (9.5) |
| Limited consultation hours at pediatrician/diabetes care center    Always    Often    Sometimes    Rarely    Never | 0 (0)  0 (0)  5 (23.8)  5 (23.8)  11 (52.4) |
| Different eating habits    Always    Often    Sometimes    Rarely    Never | 2 (9.5)  4 (19.0)  5 (23.8)  4 (19.0)  6 (28.6) |
| Different everyday structure    Always    Often    Sometimes    Rarely    Never | 3 (14.3)  13 (61.9)  3 (14.3)  0 (0.0)  2 (9.5) |
| Different sleeping patterns    Always    Often    Sometimes    Rarely    Never | 3 (14.3)  4 (19.0)  4 (19.0)  6 (28.6)  4 (19.0) |
| Different mood    Always    Often    Sometimes    Rarely    Never | 1 (4.8)  8 (38.1)  2 (9.5)  4 (19.0)  6 (28.6) |
| Diabetes management was more challenging during pandemic    Yes    No | 10 (47.6)  11 (52.4) |
